# Antigen-Specific T Cells and Cytokines Detection as Useful Tool for Understanding Immunity against Zoonotic Infections

**DOI:** 10.1155/2012/768789

**Published:** 2012-02-09

**Authors:** Annalisa Agnone, Alessandra Torina, Gesualdo Vesco, Sara Villari, Fabrizio Vitale, Santo Caracappa, Marco Pio La Manna, Francesco Dieli, Guido Sireci

**Affiliations:** ^1^Dipartimento di Biopatologia e Biotecnologie Mediche e Forensi (DiBiMeF), Università di Palermo, Corso Tukory 211, 90134 Palermo, Italy; ^2^Istituto Zooprofilattico Sperimentale della Sicilia, Via Gino Marinuzzi 3, 90129 Palermo, Italy

## Abstract

Zoonoses include a broad range of diseases, that are becoming of great interest, due to the climate changing, that cause the adaptation of vectors to new niches and environments. Host immune responses play a crucial role in determining the outcome of infections, as documented by expansion of antigen-specific T cells during several zoonotic infections. Thus, understanding of the contribution of antigen-specific T-cell subsets in the host immune response is a powerful tool to evaluate the different immunological mechanisms involved in zoonotic infections and for the development of effective vaccines. In this paper we discuss the role of T cells in some eukaryotic and prokaryotic infectious models.

## 1. Introduction

Zoonotic diseases are a significant burden on global economies and public health [1] and are due to the unaware role of wild and domestic animals, which act as reservoir or hosts of the etiological agents. More than 60% of emerging infectious diseases are constituted by zoonoses and the majority of these are increasing significantly over time [2]. In 2009 the World Organization for Animal Health (OIE) has commissioned Civic Consulting to conduct a study on the Cost of National Prevention Systems for Animal Diseases and Zoonoses, estimating that in developing and transition countries substantial differences in the public expenditure for the National Prevention System for Animal Diseases and Zoonoses exist, reaching from 10 million international dollars to 167 million international dollars [3]. The impair they cause should be attributed not only to human and animal suffering but also to the hampering agricultural production, the decreasing of food availability, and the creation of barriers to international trade [1], as well as the veterinary management, the maintenance of surveillance plans, and the capillary control in the food industry chain of production.

Many zoonotic agents are transmitted by vectors, others by contaminated water or food, and others by direct transmission. A broad range of pathogens can be responsible for zoonoses, ranging from virus to prokaryotic to eukaryotic (unicellular or multicellular), and the great difference in the antigenic input for the immune system of the hosts implies that many different branches of immunity could be involved in protection or pathogenesis.

T cells play a pivotal role in immune functions since they are able to act not only differentiating in different subsets (including *γδ* T-lymphocytes and Cytotoxic T-Lymphocytes) but also inducing the production of antibodies that inhibit the pathogen spreading, both directly and with the help of other branch of the immune system.

Homeostatic cytokines are those factors able to regulate multiplication and differentiation of many cell types; T cells are dependent on contact with IL-2, IL-7, and IL-15, for their survival and intermittent homeostatic proliferation [4]. T-helper cell differentiation is instructed by distinct environmental cytokines, that upregulate the expression of lineage-specific transcription factors and inhibit the alternate differentiation pathways [5]. The contact between the naïve T cell and the antigen induces the expression of IL-2 and IL-2 receptor leading to the entry of the T cell into several rounds of proliferation and to the differentiation in Th1, Th2, Th17, and induced regulatory T (iTreg) cells. The process consist of an intriguing cytokines puzzle, where IL-4 plays a major positive feedback role in Th2 differentiation, and IFN-*γ*, together with IL12, determines Th1 induction [6]. IL6 and IL1 are necessary for Th17 production, while the role of TGF*β* needs still to be deeper investigated [7, 8]. Finally, activated naïve CD4 T cells stimulated by TGF-*β* in the absence of proinflammatory cytokines develop into iTreg cells [9].

The complex network of cytokines function is resolved in a balance from different T-cell activation pathways (Th1/Th2, Th1/Treg, Th2/Treg, Th1/NK, and/or *γδ* T cells). Although T-cell-mediated immune response during zoonotic infections is poorly studied, the facilities in the setting-up experimental conditions make it good system for a deeper investigation on the specific activation of T-lymphocytes.

It is well known that protozoan, helminthic parasites, and intracellular bacteria are able to survive within the host, in spite of the activation of both innate and adaptive immune response [10]. Zoonotic infections caused by eukaryotic organisms are intriguing systems where the antigen-specific T-cell expansion can be studied [11].

**Figure 1:**
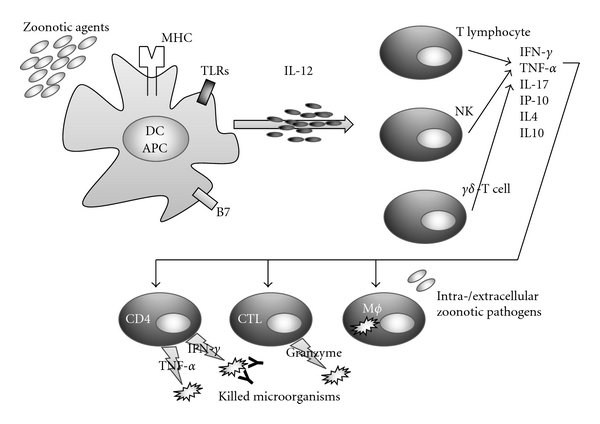
Schematic network of cells and molecules in response to zoonotic agents. An “oversimplified” scenario constituted by various cells and molecules involved both in binding of epitopes derived from pathogens and in the effector mechanisms hereby represented. APCs bind zoonotic derived epitopes and present them to various types of lymphocytes, in the context of MHC molecules and/or Toll-Like Receptors (TLRs). These subsets, producing different cytokines, could activate effector “protective” mechanisms involving macrophage killing, cytotoxic activity by CTL and/or CD4, and release of various cytokines, thus leading to the damaging of zoonotic pathogens. The killing by CTL, that could be not only CD8 but also NK cells, could be also due to an ADCC phenomenon with the contribution of antizoonotic epitopes,-specific antibodies.

Helminthes have the ability to drive the differentiation of naïve CD4 T cells to the Th-2 subset of effector cells which are able to eliminate the pathogens by the actions of antibodies induced by Th2 cytokines. During a protozoarian infection, protozoa are usually phagocytosed into macrophages, previously activated by Th1 lymphocytes, and are able to survive evading host immune response. As it happens in the case of intracellular bacteria, infected cells loose the ability to kill the pathogen, and Cytotoxic T-Lymphocyte- (CTL-) mediated immune response is needed for the elimination of microorganisms into macrophages [12] (Figure 1). The naïve T cells encounter the antigen in the peripheral lymph node, develop toward effector cells, and migrate to the site of infection for the killing of infected cells. This process is finely tuned by cytokines cross-talk and microbial ability to evade host immune response.

B cells and humoral response play the main role in the clearance of extracellular bacteria. Nevertheless, a certain enrolment of T-cells has been demonstrated [13]. In this paper, we draw attention on different mechanisms of T-cell-mediated immunity, in order to compare the mechanisms of immune modulation induced by various zoonotic agents.

## 2. T Cells and Cytokines Induced by Eukaryotic Zoonotic Agents

The nematode parasites *Toxocara (T.) canis *and* T. cati* choose dogs and cats as definitive hosts, respectively. Sometimes, when embryonated eggs are accidentally ingested by humans, larvae hatche in the small intestine, penetrate the intestinal wall, and cause the larva migrans syndrome [14]. Toxocariasis symptoms are classified according to the organs affected in visceral larva migrans (VLMs) and ocular larva migrans (OLMs). In the latter toxocariasis pathological effects on the host are restricted to the eye and optical nerve [15], while in the case of VLM, symptoms can persist for more than one year and include abdominal pain, coughing, headache, and normal or mildly elevated eosinophilia [16]. A recent survey [17] emphasizes that the seroprevalence value among humans is considerably high, thus demonstrating the relevance of this pathology. *T. canis* is able to control host immune response, through the modulation of cytokines produced by immune cells. The immunomodulatory effect has been demonstrated in mice, where the stimulation of normal macrophages with *T. canis* antigen *in vitro* induced IL-1*α*, IL-6, IL-10, and TGF-*β*, but not IL-12 and TNF-*α* [18]. Prototypical immune responses are characterized by increased lymphoproliferation of CD4^+^ and CD8^+^ T cells, increased production of IL-4 and IL-5, eosinophilia, and augmented production of IgE, as previously described in humans and mice [13–15]. As regards the immune response in dogs, it has been demonstrated that *T. canis* is able to induce antigen-specific IFN-*γ* production in pregnant dogs and in their puppies [19]. Blood mononuclear cells (BMCs) were isolated from pregnant dogs and their puppies and were cultured in the presence of ESAg (Excretory/Secretory Antigen of *T. canis*). Cytokine levels were tested in cultures' supernatants by ELISA, and it was noted that IL-10 concentration increases during pregnancy in infected animals while IFN-*γ* production decreases. On the contrary IL-10 concentration decreases with the age of infected puppies while IFN-*γ* amount increases. It appears clear that immune cells of infected dogs undergo *T. canis*-induced modifications. These modified pattern of cytokines detected in *T. canis* could be due to a synergistic effects of physiological changes of immunity during pregnancy and in the first month of life, and/or direct effects mediated by parasite interaction with host immunity. The finding that IL-10 and IFN-*γ* levels were significantly modified in infected pregnant dogs and their puppies provides new perspectives for immunotherapeutic interventions based on switch of Th2 to Th1 cytokine pattern in females before pregnancy.

Another system to understand the role of T cells in eukaryotic zoonotic infections is echinococcosis. Alveolar echinococcosis is caused by the metacestode stage of *Echinococcus multilocularis*. The definitive hosts are the foxes, which release Echinococcus eggs in the foecal matter, spreading them in the environment. Little rodents acquire the infection by ingesting eggs and carry the infection in their liver. Humans are aberrant intermediate hosts [20]. In humans, metacestode stage of the worm affects the liver, where an abdominal mass develops; other symptoms may arise like abdominal pain, jaundice, and liver failure [21]. The severity of the disease is dependent on the genetic background of the host and on the balance between the Th1-related immune response, associated with protection, and the induction of the immune tolerance by the parasite itself [22]. In experimentally infected C57BL/6J mice the promotion of the disease seems to be associated with the expansion of different T-cell subsets: spleen cells harvested at different time points after infection were stimulated *in vitro* with a crude parasite extract. A strong CD4^+^ proliferative T-cell response was observed at the early stage of infection, and IFN-*γ*, IL-2, and IL-5 were produced within the first weeks after infection whereas the detection of IL-10 was slightly delayed [23]. Cystic echinococcosis is caused by *E. granulosus*. The main domestic cycle is maintained between dogs and sheep, with man as accidental intermediate host. The disease is acquired by ingesting eggs, originating from the faeces of definitive hosts (dogs, wolves, and other carnivores) [24], and it typically affect, the liver. It is often asymptomatic, but in case of rupture of the cyst, secondary infection and anaphylactic reaction can occur. The most frequent complications are pain, obstructive jaundice, cholangitis and sometimes shock [25]. It has been demonstrated that a restimulation of PBMC from affected patients with the crude antigen induces an upregulation of IL-5 and IL-10 [26] as well as a downregulation of IL-1 and TNF-*α* mRNAs [27].

The opportunistic parasite *Toxoplasma gondii* belongs to the phylum apicomplexa. Feline acts as definitive hosts in its life cycle, while mammalians, including humans, are intermediate hosts. Human toxoplasmosis is usually asymptomatic or paucisymptomatic, but the parasite is able to cross the intestinal barrier and disseminate through the body, reaching muscle, central nervous tissues, eyes, and placenta [28]. Congenital toxoplasmosis may hesitate in retinochoroiditis and/or mental abnormalities [29].

The infection by* T. gondii* induces a strong cellular response essential for the host resistance [30]. In particular, it has been noted since 1990 that upon an *in vitro *stimulation with *T. gondii* antigen, a strong CD8^+^ T-cells response, sustained also by CD4^+^cells expansion, is mounted [31]. The role of CD4^+^ in the activation of CD8^+^ has been demonstrated in mice [32], where the generation of optimal numbers of antigen specific CD8^+^ effector T cells was found to require CD4^+^ T-cells help. The parasite is also able to induce a strong natural killer (NK) cells activation and macrophages production of IL-12, both ending in a massive IFN-*γ* production. The IFN-*γ* production is sustained by *γδ*-T lymphocytes [33] that help CD4^+^ and CD8^+^T cells to restrict parasite growth until the emerging of the complete adaptive response. It has been recently demonstrated that the CD8^+^ T-cells response is sustained both by “homeostatic cytokines” IL-15 and IL-7 and that the absence of IL-15 or IL-7 alone does not affect CD8^+^ T cell activation during acute toxoplasmosis [34], thus suggesting that these cytokines could act in synergy. Immune response of congenitally infected newborns to *T. gondii* undergoes to a process that leads to anergy [35, 36], probably due to a developing immune system of the infant. In this case, both *αβ*- and *γδ*-T cells become unresponsive when stimulated with *T. gondii*-specific antigen. Nevertheless, V*δ*2^+^ *γδ* T cells are able to lose tolerance before *αβ*-T-cells, and to confer protection against the chronic phase of infection in congenitally infected children [37]. Indeed, *γδ* T cells are considered to undergo peripheral tolerance, thus persisting in blood longer than *αβ* T lymphocytes which are deleted in the thymus during* T. gondii* infection [37].

A useful model to better understand immune response to eukaryotic zoonotic agents is constituted by Leishmaniasis and its related immunity. Leishmaniasis is a vector-borne disease caused by obligate intramacrophage protozoan parasite of the genus Leishmania and its incidence is increasing in nonendemic areas due to changing patterns of international travel and to population migration [38]. Visceral leishmaniasis (VL) or kala-azar is one of several diseases caused by more than 20 species of the protozoan parasite Leishmania. The infection tends to affect mainly children, but immunosuppression and HIV increase the possibility to contract the illness. The common symptoms are fever, malaise, shivering or chills, weight loss, anorexia, and discomfort in the left hypochondrium [39]. In experimental *L. major* infections genetically resistant mice develop a T-cell response dominated by a CD4^+^ (Th1) phenotype characterized by IFN-*γ* secretion while in susceptible mice the dominant response is a CD4^+^ (Th2) phenotype characterized by interleukin IL-4, IL-5, and IL-13 secretion [40]. These observations of *L. major* in mice led to the emergence of the Th1/Th2 paradigm as opposing cytokine responses in the control of infections [41, 42]. The balance of Th1 to Th2 responses determines the outcome to infection. In the natural disease both Th1 and Th2 cellular subtypes are activated. Resistance to infection depends on production of cytokines such as IFN-*γ*, TNF, IL-2, and IL-12. These cytokines stimulate cell-mediated immunity which eliminates the infection activating leishmanicidal activity of macrophages [41, 42]. The infection in dogs shows different clinical presentations, from subclinical/asymptomatic to a fully developed disease, depending on the host's immune responses. The Th1/Th2 dichotomy is not clear in the different forms of canine leishmaniases, because it depends on physiological status of the infected subject. The production of IL-4, IL-5, IL-6, and IL-10, which in turn promote B-cell proliferation and antibody production, is the cause of susceptibility of dogs, which become not able to control the infection [43–45]. Our experience is focused to evaluate cytokine expression level with a quantitative real-time PCR assay to measure expression levels of cytokines relative to either Th1 or Th2 patterns in the blood of naturally infected asymptomatic dogs. High expression levels of IL-2 and IFN-*γ* were detected at the first observation, which decreased over time. Opposite cytokine-based effects were detected in infected dogs. In those that had a clinically evident outcome, IL-2 and IFN-*γ* were initially not expressed, but their levels suddenly increased with the appearance of clinical signs [43]. Furthermore from our study it was confirmed that IL-12 represents a marker of active disease, while IL-18 cannot be involved in the progression from asymptomatic to active disease. These data suggest that response to Leishmania in the dog does not fit into a specific cytokine profile.

## 3. Antigen-Specific T Cells and Derived Cytokines Detection in Prokaryotic Infections

Among prokaryotic microorganisms able to cause zoonotic disease, Leptospira, Brucella, and Mycobacteria offer suitable models to analyze the role of immune response against these pathogen since the related immunity could involve different antigen-specific T cell subsets. *Leptospira interrogans* is one of the main causative agents of leptospirosis. The pathogen is able to persist in the kidneys of infected (wild and domestic) animals and is spread in the environment through their urine. It is transmitted to humans through skin abrasions and causes haemorrhage, diarrhoea, renal impairment, and aseptic meningitis [46]. Phagocytosis is the main process that allows the clearance of the pathogen, and it has been recently demonstrated that the bacteria undergo a complex transcriptional regulation in order to evade host immune response [47]. In particular they downregulate the major OMPs (Outer Membrane Proteins) through the action of a hypothetical transcriptional factor. It is well accepted that humoral immunity has an important role for the elimination of extracellular bacteria, but sometimes antibodies alone could not be sufficient, especially in the case of *L. borgpetersenii* serovar Hardjo [48]. In this and other cases, IFN-*γ* plays an important role for the activation of macrophages and the production of IgG2 class of immunoglobulins [49, 50]. The involvement of a cellular immune response has been recently demonstrated: a strong Th1 response was recorded by the observation of the IFN-*γ* production following the *in vitro* stimulation of vaccinated bovine PBMC with the specific antigen [51]. The results from vaccinated animals indicated that approximately two-thirds of IFN-*γ* ^+^ cells were within the CD4^+^ T-cell population while the remaining one-third were *γδ* T cells [51]. Furthermore, Guo et al. have recently reported the existence of specific cytotoxic CD8^+^ T cells in patients with leptospirosis and have detected a potential epitope of the leptospiral protein LigA, able to elicit specific cytotoxic T-lymphocyte (CTL) responses [13]. Naiman and Guo suggest that Th1 response to Leptospira requires the cooperation between two or more T cell subsets like *γδ*, CD8^+^, CD4^+^, and so forth. In Leptospira-infected hamsters a new soluble factor was shown to be important for the protection: IP-10 [52]. This evidence points to T cell-derived chemokines in zoonosis. These proteins are able to induce cell migration from lymphoid organs to affected tissues and they are also considered markers of T cell maturation [53]. Indeed, future approaches for a deeper analysis of T cell response in zoonoses could be comprehensive of the characterization of the released chemokines and their receptors.

A very hot field in veterinary immunology is represented by T cell responses against intracellular bacteria. Tuberculosis and Brucellosis remain major worldwide health emergencies among zoonotic bacterial infections, and a better understanding of the host immunological reactions to these pathogens is fundamental for improving both therapies and vaccines strategy, as well as to prevent dissemination of the infectious agents in the herds. Tuberculosis causes in host mild fever and a wide range of symptoms depending on the localization of the Mycobacterium (pneumonia, kidney failure, meningitis especially in children, etc.) [54].

Animal tuberculosis is mainly observed in cattle (less frequently also in horses, swine, dogs, cats, sheep, and goats), caused by *Mycobacterium (M.) bovis*, and in birds, due to *M. avium*. Human tuberculosis is mainly caused by *M. tuberculosis*, but around 10% of total infections are due to *M. bovis*, typically as professional disease, while *M. avium* can cause disease in immunodeficient patients [55]. Dogs and parrots are highly susceptible to *M. tuberculosis* by the contact with infected humans. T-lymphocytes play a central role in the control of *M. tuberculosis* replication, as this infection evokes a strong cell-mediated immune response. Protective immunity against *M. tuberculosis* is due to adaptive cellular immune responses, and protective immunity correlates to the induction of T cell cytokines following antigen specific stimulation. CD4^+^ and CD8^+^ T cells are key components of anti-mycobacterial immunity [56, 57]. Both IFN-*γ* production and cytotoxic activity against infected target cells contribute to bacteria killing with lysis of infected cells [58, 59].

T cells response after *in vitro* stimulation of human PBMCs with *M. tuberculosis-*specific antigens (e.g., Purified Protein Derivative, or PPD) can be assessed by measuring intra- and extracellular IFN-*γ* [60]. The severity of *M. tuberculosis* infection may be detected by measuring CD4^+^ and CD8^+^ T cells, as their numbers markedly decrease in patients with severe tuberculosis, which can be a sign of suppressed cellular immunity in these patients [60]. Particularly, patients with active TB have a lower number of both CD4^+^ T cells and their naïve, effector, and late differentiated memory subsets [61], with a drop in all the three phenotypic populations. Similarly, CD8^+^ T cells counts were also significantly different between infected and negative patients. At least partially, these disturbances seem to be restored to baseline after successful therapies [61].

In our experience with cattle [62] it has been showed that cocktails of epitopes from ESAT-6 (the 6 kDa early secretory antigenic target of *Mycobacterium tuberculosis*) are recognized with high frequency by CD8^+^ T lymphocytes of naturally infected cattle, thus confirming a role of ESAT-6 specific CD8^+^ T cells in the response to *M. bovis*. Nevertheless, the number of IFN-*γ*-positive CD8-negative cells was larger than that of IFN-*γ* ^+^ CD8^+^ T cells, indicating that IFN-*γ* ^+^ CD8^+^ T cells are not the dominant subset responding to stimulation with ESAT-6-derived peptides. Nevertheless, ESAT-6-specific T-cell expansion could be useful to detect the early phase of the disease thus limiting the dissemination of *M. bovis*.

Other cytokines such as TNF-*α*, IL-2 [63], MCP-2 [64], and IP10 [65] were shown to be involved in the anti-mycobacterial immune responses in humans; Th1- and other cytokines interacting with macrophages are commonly considered as mediators of anti-mycobacterial biological agents. When reagents for the detection of these cytokines in vertebrates will be available, it could be intriguing to understand the role of these cytokines in mycobacterial immune response also in veterinary infections.

Brucellosis is a multisystemic disease with a broad range of symptoms, usually beginning with acute febrile illness, headache, malaise, and myalgia. Gastrointestinal signs as vomiting, anorexia, and nausea may also occur [66]. Humans are susceptible to *Brucella (B.) suis*, *B. Abortus,* and *B. canis*, and, more frequently, to *B. melitensis*. The disease can be transmitted by both direct and indirect contact with infected animals or secretions, or by eating contaminated food (especially unpasteurized milk and fresh cheeses). Interhuman transmission is extremely rare [67].

Brucella invades and proliferates within monocytes. In addition to the central role of monocytes/macrophages, other cells of the innate immune response are recruited and influence the interaction between bacteria and host. For instance, human V*γ*9V*δ*2 T cells play an important role in the early response to infection [67], and their number dramatically increases in the peripheral blood of patients with acute brucellosis [68], reaching 30% of the total T lymphocytes. V*γ*9V*δ*2 T cells are specifically stimulated by Brucella to secrete TNF-*α*, important for the autocrine activation of macrophage functions, IFN-*γ*, and other cytokines [69]. *In vitro*, V*γ*9V*δ*2 T cells exhibit a strong cytotoxicity against Brucella-infected cells. V*γ*9V*δ*2 T cells decrease the development of intracellular Brucella releasing lytic granules and/or acting through Fas-mediated signals to lyse infected macrophages. It was also shown that the recruitment of NKG2D by its ligands is sufficient to induce cytokine production and the release of lytic granules thus increasing the TCR-triggered responses of V*γ*9V*δ*2 T cells. The interaction between NKG2D and its main ligand expressed on Brucella-infected macrophages, UL16-binding protein 1 (ULBP1), is involved in the inhibition of bacterium development [69]. As demonstrated in the case of V*γ*9V*δ*2 T cells, it was shown that also NKT cells are able to exert an anti-Brucella *in vitro* activity, either secreting cytokines or killing infected macrophages [70]. NKT and V*γ*9V*δ*2 are considered as quite unrestricted T cells as they do not recognize MHC and peptides, but they expand following stimulation with nonpolymorphic MHC-like molecules CD1 and/or with nonpeptidic and glycolipid ligands. A cross-talk between V*γ*9V*δ*2 and NKT, due to cytokines released in the milieu, could be responsible for the activation of NKT in synergy with a possible upregulating role of CD1 molecules expression exerted by Brucella antigens. The previously described subsets activated during Brucella infection could exert a protective role during Brucella infection through their potent cytotoxic activity.

## 4. Concluding Remarks

Each microorganism hereby evaluated elicits a particular type of immune response. A “classical” Th1-mediated protective immune response was detected during zoonotic infections like leishmaniasis or tuberculosis. Toxoplasma-, Brucella- and Leptospira-induced immune response involves a wide range of T cells including *γδ* and NKT cells. The *in vitro* and *ex vivo* detection of T cells upon stimulation with the specific antigen allows going insight in the host/pathogen interaction. The equilibrium established after such dialogue is critical for the further ongoing of the infection. A complex network of T cells, cytokines, and chemokines could be studied to better understand the interactions between zoonotic agents and receptors of innate and adaptive immunity. This tool could be useful to develop vaccines and immunotherapies in the next future.
